# Valuable Remedies in Diphtheria

**Published:** 1889-05

**Authors:** E. S. Richardson

**Affiliations:** Reed City, Mich.


### VALUABLE REMEDIES IN DIPHTHERIA.

BY E. S. RICHARDSON, M. D., REED CITY, MICH.

After an extended experience in the treatment of diphtheria, both sporadic and epidemic character in which I lost many of my cases when I followed the treatment recommended by the authors of several standard text-books on medicine, I finally settled upon the following remedies the effect of which has been most gratifying, viz : First, alcohol in full strength, used topically. In cases where the membrane had invaded the posterior nares, just such cases when with other remedies they invariably proved fatal, alcohol saved every patient in which it was used ; the morbid action was immediately arrested and convalescence rapid. In asthenic cases, and they are sure to be such if not early arrested, I find marked benefit from spirits of turpentine in stimulating doses, followed by tonic doses of quinine. Dr. Mackenzie, of London, England, extolls the beneficial action of an alcoholic solution of shellac, but I believe the medical virtue is due to the alcohol and not to the "protection of the surfaces by the thin film of gum shellac." I think it must be conceded that alcohol is one of our best germicides, and is antiseptic par excellence. Armed with these remedies I no longer dread to approach this disease, but do so with the greatest confidence of success in all cases that are not already moribund.—*Medical Summary.*

